# Task‐dependent intermuscular coherence between postural muscles during voluntary upright reaching

**DOI:** 10.1113/EP093222

**Published:** 2026-03-10

**Authors:** Imma Ceriello, Riccardo Borzuola, Valentina Camomilla, Andrea Macaluso, Madeleine Lowery

**Affiliations:** ^1^ Laboratory of Bioengineering and Neuromechanics, Department of Movement, Human and Health Sciences University of Rome ‘Foro Italico’ Rome Italy; ^2^ School of Electrical and Electronic Engineering University College Dublin Dublin Ireland

**Keywords:** common input, EMG–EMG coherence, intermuscular coherence, postural control, upright reaching

## Abstract

Intermuscular coherence provides a window into the neural mechanisms coordinating posture and movement. This study investigated task‐dependent modulation of coherence between postural muscles in healthy young adults performing upright forward and lateral reaching tasks. Bilateral electromyographic activity was recorded from trunk and ankle muscles from both the dominant and non‐dominant reaching sides. Coherence was estimated in the delta, alpha, beta and low gamma frequency bands. During forward reaching, delta‐band coherence was higher than in lateral reaching across bilateral homologous muscles and trunk–limb pairs within the posterior chain (all *P* < 0.001, *g* ≈ 1.765–3.712). Conversely, during lateral reaching, the non‐dominant ankle antagonist pair exhibited higher delta‐band coherence (*P* < 0.001, *g* ≈ 2.521–2.601) and increased beta/low gamma‐band coherence (*P* < 0.05–0.001, *g* ≈ 0.860–1.040). In this pair, delta‐band coherence of this antagonist pair correlated negatively with centre‐of‐pressure path length (*r* = −0.707, *P* = 0.0456). On the dominant side, delta‐ and beta‐band coherence correlated positively with co‐contraction (*r* ≈ 0.680–0.745, *P* ≈ 0.0319–0.00730). The ankle agonist pair exhibited greater delta‐band coherence than antagonists (*P* < 0.001, *g* ≈ 1.583–3.064) and minimal variation in beta and low gamma bands, consistent with their synergistic role in postural control. These findings demonstrate that coherence organization adapts to postural demands: forward reaching engages bilateral and posterior‐chain coupling for sagittal stability, whereas lateral reaching elicits asymmetric, limb‐specific strategies combining automatic and voluntary components. This modulation highlights the adaptability of neural control processes that regulate muscle coordination under varying mechanical demands.

## Abstract (highlights)

- **What is the central question of this study?**
  How does the central nervous system (CNS) modulate intermuscular coherence across different lower‐limb and trunk muscle pairs to meet the postural demands of directional reaching tasks?
- **What is the main finding and its importance?**
  Intermuscular coherence during upright reaching varies with direction and muscle pair. Delta‐band coherence supports automatic coupling, in bilateral, intra‐limb synergistic and trunk–limb muscles during forward reaching. In contrast, beta‐ and low gamma‐band coherence reflect cortical involvement in voluntary modulation during unilateral and asymmetric conditions. Lateral reaching revealed side‐specific strategies. These findings highlight flexible, frequency‐ and task‐specific CNS mechanisms coordinating postural synergies.

## 1 INTRODUCTION

Maintaining balance during voluntary whole‐body movements is a crucial role of the postural control system. Tasks such as forward and lateral reaching (Brauer et al., 1999; Duncan et al., 1990) performed from a standing position involve intentional shifts in body weight, present heightened postural challenges and require precise neuromuscular coordination. These tasks are commonly utilized to investigate the neural mechanisms of postural control (Hilt et al., 2018; Leonard et al., 2009; Maranesi et al., 2016) and to evaluate dynamic postural control in clinical settings (Berg, 1989; Haines et al., 2007; Rose et al., 2006), as they provide an ecologically, functionally relevant and experimentally controllable framework to study the neural strategies involved in postural control. Unlike static standing, reaching while upright introduces dynamic perturbations to balance control that require coordinated adjustments across multiple joints. This results in dynamic interactions between cortical and spinal neural networks to coordinate body segments and maintain stability. The central nervous system (CNS) plays a fundamental role in preserving postural stability during these tasks by coordinating precise adjustments in muscle activation throughout the trunk and lower limbs. To understand how the CNS exerts control over such actions, a key concept in the development of motor control is the idea that the CNS organizes the complex neuromuscular system by integrating motor components into functional or synergistic groups (Bernshteĭn, 1967). Within this framework, muscle synergy theory has been proposed as a means by which the CNS simplifies motor control (d'Avella & Bizzi, 2005). Muscle synergies serve as a library of motor subtasks that the CNS can flexibly combine to facilitate the execution of complex and natural movements (d'Avella & Bizzi, 2005; Torres‐Oviedo & Ting, 2007). It has been suggested that correlated neural input might serve as the mechanism through which the CNS organizes the activation of muscles that work together as a synergistic muscle group (Boonstra et al., 2009; Danna‐Dos‐Santos et al., 2014; Degani et al., 2017; Farmer et al., 2007). This stems from the idea that synchronizing neural oscillations is how the CNS accomplishes extensive integration among its cortical and subcortical components, including those that play a role in the generation and regulation of movement. Intermuscular coherence (EMG–EMG coherence) offers a framework to examine synchronized activity between two electromyographic signals in the frequency domain (Farmer et al., 1993) and can yield insights into shared neural inputs: identifying coherence within specific frequency bands allows inference about the origin and functional role of common neural drives that differ according to the functional demands of the task (Baker et al., 1997). Thus, it provides an indirect assessment of such shared common drive, serving as a valuable method to assess oscillatory coupling and neural synchronization. Intermuscular coherence is typically characterized within four main frequency bands, each associated with specific neural processes. Low‐frequency oscillations ranging from 0–5 Hz, defined as the delta band, are associated with the common modulation of motor unit mean firing rates and muscle force production, thus likely reflecting co‐modulation of muscle activity (Boonstra et al., 2009; De Luca & Erim, 1994; Kamen & De Luca, 1992; Lowery et al., 2007; Myers et al., 2004). Oscillatory activity within the alpha band (8–12 Hz) has been linked to physiological tremor (Budini et al., 2014; McAuley & Marsden, 2000) and the engagement of the corticoreticulospinal pathway (Baker & Baker, 2003; Charalambous et al., 2024; Conway et al., 1995; Grosse & Brown, 2003). Higher frequency oscillations in the beta (13–35 or 15–30 Hz) and low gamma (35–60 or 30–60 Hz) frequency ranges are related to the functioning of the transcortical pathway and reflect shared corticospinal inputs to the motoneuron pool (Charalambous et al., 2024; Conway et al., 1995; Fisher et al., 2012).

Building on this physiological framework, several studies have applied intermuscular coherence to various postural control tasks, including static stance, unipedal tasks, and forward leaning, providing insights into how the CNS adapts to varying levels of balance challenge under different conditions and organizes the activation of muscles (Danna‐Dos‐Santos et al., 2014; García‐Massó et al., 2016; Konieczny et al., 2022; Nandi et al., 2019; Noé et al., 2017; Nojima et al., 2020; Tsiouri et al., 2024; Watanabe et al., 2018a, 2018b). Together, these studies show that coherence is sensitive to task difficulty and reflects functional neural coordination that varies across postural and motor behaviours. The majority of studies to‐date have focused on trunk and lower limb muscles, particularly ankle plantar‐ and dorsiflexor muscles, given their role in maintaining postural stability and controlling body sway (Danna‐Dos‐Santos et al., 2014; Noé et al., 2017; Watanabe et al., 2018a, 2018b). Robust coherence has been reported between bilateral/unilateral plantar flexor muscles and between agonist–antagonist pairs (e.g., tibialis anterior and plantar flexors) (Boonstra et al., 2015; Degani et al., 2020; Nandi et al., 2019; Noé et al., 2017; Nojima et al., 2020; Watanabe et al., 2018a, 2018b). Coherence between trunk and lower limb muscles has also been observed, underscoring the integration of proximal and distal muscles in balance control (Danna‐Dos‐Santos et al., 2014; Degani et al., 2017; Noé et al., 2017).

While intermuscular coherence has been investigated during isolated postural tasks, compound movements involving dynamic transitions from quiet standing to perturbed or destabilized postures remain largely unexplored. Evaluating intermuscular coherence during these movements could provide key insights on the neurophysiological adaptation of postural control in voluntary movements entailing sudden changes and movement initiation under unstable conditions. In a previous study, we demonstrated that muscle synergies underlying multidirectional upright reaching tasks are directionally tuned, although some patterns are shared across different directions (Ceriello et al., 2026). This indicates that, although the CNS reconfigures muscle activation according to biomechanical constraints, there is a common organizational framework.

The present study expands upon this concept by exploring the neural mechanisms that support the flexible recruitment of these task‐specific yet partly shared muscle synergies. Specifically, we aimed to determine whether the coherence of oscillatory activity between postural trunk and lower‐limb muscles reflects direction‐dependent modulation or a common neural drive across reaching directions. Moreover, we evaluated how these neural coordination patterns interact with the biomechanical requirements of balance control, to provide a comprehensive view of the interplay between neural and mechanical aspects of postural regulation. Understanding how neural coupling adapts, or fails to adapt, to distinct postural demands provides key insight into the flexibility of CNS control and may constitute a physiological marker for impaired postural adaptability in clinical populations.

We hypothesized that intermuscular coherence would vary based on the direction of reaching, demonstrating task‐specific neural adaptations to the biomechanical requirements of different directions. Additionally, we hypothesized that functionally synergistic muscles would show synchronized activity, suggesting a common neural drive to postural stability. Although specific hypotheses were formulated, the analysis of intermuscular coherence in these multi‐segmental postural tasks retains an exploratory component, given the limited previous evidence on such conditions.

## 2 METHODS

### 2.1 Ethical approval

The study received approval from the University Research Committee of the University of Rome ‘Foro Italico’ (CAR 154/2023) on 4 May 2023, subsequently revised on 23 April 2024 (CAR 154/2023/Rev). Prior to data collection, all participants provided written informed consent, and the study was conducted in accordance with the *Declaration of Helsinki* as updated in 2024 (World Medical Association, 2025).

### 2.2 Sample population

Seventeen healthy young participants without any neurological or musculoskeletal disorders that might influence mobility took part in the study (10 males and 7 females; average age: 27.5 ± 3 years; average height: 1.7 ± 0.1 m; average body mass: 64.9 ± 8.8 kg). Among the participants, there were two left‐handed individuals and 15 right‐handed individuals, with handedness assessed using the Edinburgh Handedness Inventory (Veale, 2014). The sample size was chosen based on previously published research in which intermuscular coherence analyses were employed (García‐Massó et al., 2016; Kattla & Lowery, 2010; Watanabe et al., 2018a).

### 2.3 Experimental protocol

Participants were barefoot and positioned with feet hip‐width apart to ensure a stable base of support. Adhesive tape was used to mark foot placement for consistency throughout the trials. Participants were asked to complete three sets of tasks: unilateral functional reach (uniFR), bilateral functional reach (biFR) and lateral reach (LR) at a comfortable pace of their choosing. Each set comprised 10 repetitions where participants aimed to extend their reach as far as possible without stepping, raising their heels or losing balance. The tasks were conducted in a freestanding manner, away from any walls, following the recommended guidelines for functional reach (FR) execution (Duncan et al., 1990). In the uniFR and LR tasks, participants utilized only their dominant arm, while the biFR required reaching forward with both arms simultaneously. These task variations were selected to examine how different coordination demands influence postural control. The unilateral tasks were executed with the dominant limb to emphasize direction‐specific control strategies, as anterior–posterior and medial–lateral movements involve distinct biomechanical demands and muscle activation patterns to maintain balance. The bilateral task, instead, was included to assess whether engaging both limbs during anterior–posterior reaching would modify intermuscular coherence patterns through greater intersegmental coordination (Ceriello et al., 2026).

Each repetition began with participants lifting their arm or arms, contingent on the task, and extending their reach as far as they could while maintaining their balance. After reaching, they returned to the starting position with their arm(s) still raised before lowering them back to their sides. Participants were asked to avoid twisting their torso, bending their knees or lifting their heels. To alleviate fatigue, participants were given 2 min of rest between task sets, and the sequence of the blocks was randomized for each participant. During the activities, a research assistant was present to supervise the process.

**FIGURE 1:**
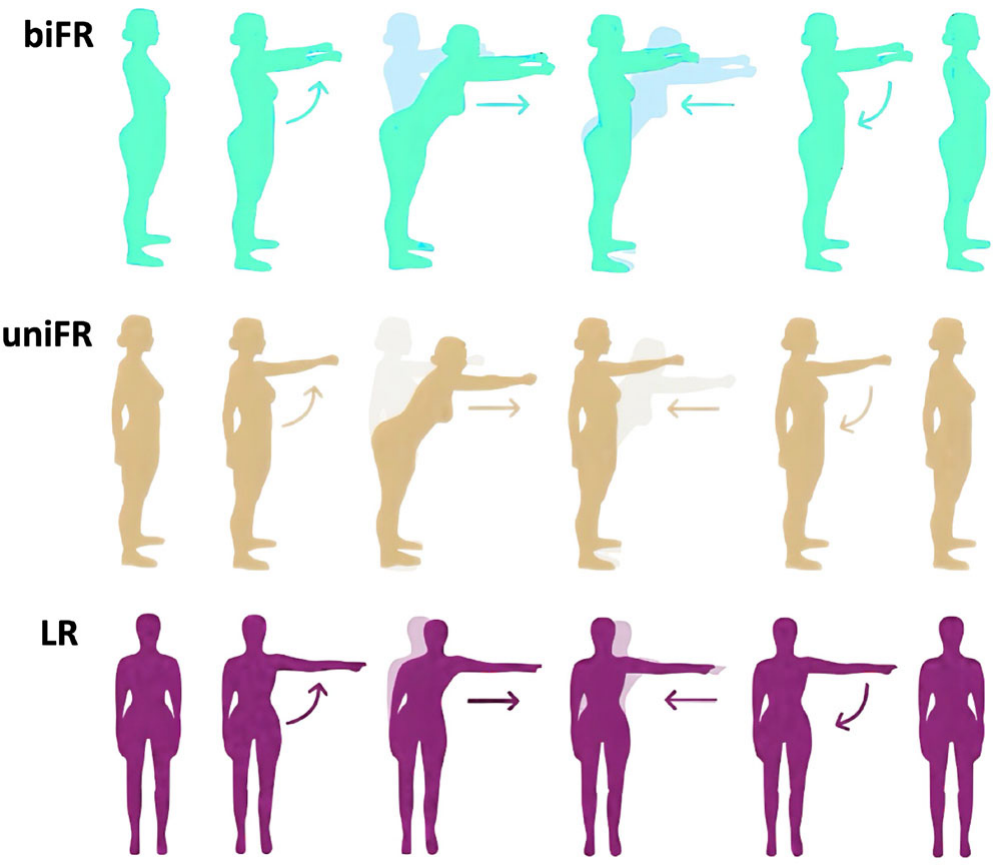
Schematic representation of the three different upright reaching tasks adapted from Ceriello et al. (2026).

A schematic representation of the given tasks is shown in Figure 1.

### 2.4 Data acquisition

Kinematic data were collected at a frequency of 200 Hz using a 3D motion capture system (VICON®, Oxford Metrics, Oxford, UK) equipped with nine infrared cameras. A total of 61 infrared reflective markers, each measuring 14 mm in diameter, were employed in accordance with the Plug‐in Gait Full Body and Convolutional Gait Model 2.5 kinematics models. Additionally, two force platforms (1 kHz, Bertec, Columbus, OH, USA) were employed to acquire dynamic data.

A wireless surface EMG system (MiniWave, Cometa, Bareggio, Italy) was used to record muscle activity from eight different muscles (four muscles on each side of the body) at a sampling frequency of 2 kHz. The EMG electrodes were attached to cleaned, abraded and properly prepared skin surfaces to assess the muscle activity of the following muscles: erector spinae (ES), tibialis anterior (TA), gastrocnemius medialis (GastM) and soleus (Sol). As the task was designed to assess dynamic balance rather than upper‐limb performance, coherence analyses were limited to trunk and lower‐limb muscles primarily contributing to postural stabilization. This selection is consistent with previous studies on postural tasks investigating intermuscular coherence patterns (Boonstra et al., 2009; García‐Massó et al., 2016; Konieczny et al., 2022; Nandi et al., 2019; Watanabe et al., 2018a). Probe placement adhered to the guidelines established by SENIAM (Surface Electromyography for the Non‐Invasive Assessment of Muscles) (Hermens & Merletti, 1996).

All the data acquisition systems were hardware synchronized.

### 2.5 Data analysis

#### 2.5.1 Identification of reaching repetitions

Kinematic data were low‐pass filtered using a fourth‐order Butterworth filter with a cut‐off frequency of 12 Hz. The velocity of the hand, represented by the marker on the middle knuckle, was calculated and then used to identify each repetition of the FR. The start of movement was defined as the point at which the hand's velocity (in the forward direction along the *y*‐axis and in the lateral direction along the *x*‐axis) reached 10% of its peak speed, while the end of the movement occurred when the hand's velocity decreased below 10% of its peak speed (Marchesi et al., 2021). Visual inspection of task execution was performed a posteriori, and those repetitions which exhibited an incorrect execution of the reaching task were removed from further analysis.

#### 2.5.2 Centre of pressure

The center of pressure (CoP) was calculated using data from two force plates corresponding to the left $\mathrm{Co}P_{L}$ and right $\mathrm{Co}P_{R}$ sides. Vertical ground reaction forces ($F_{Z}$) were obtained for each plate and used to compute a weighted average of the individual CoPs, resulting in a global CoP trajectory. A zero‐lag, fourth‐order Butterworth low‐pass filter with a cutoff frequency of 12 Hz was applied to remove high‐frequency noise. The global CoP in both the antero‐posterior (AP) and medio‐lateral (ML) directions was estimated at each time point using a weighted average based on the vertical ground reaction forces (Exell et al., 2011):

(1) $$\mathrm{Co}P_{\mathrm{AP}}\;\left(t\right)=\frac{\mathrm{Co}P_{L,y}\left(t\right)\cdot F_{Z,L}\left(t\right)+\mathrm{Co}P_{R,y}\left(t\right)\cdot F_{Z,R}\left(t\right)}{F_{Z,L}\left(t\right)+F_{Z,R}\left(t\right)}\;$$

(2) $$\mathrm{Co}P_{\mathrm{ML}}\;\left(t\right)=\frac{\mathrm{Co}P_{L,x}\left(t\right)\cdot F_{Z,L}\left(t\right)+\mathrm{Co}P_{R,x}\left(t\right)\cdot F_{Z,R}\left(t\right)}{F_{Z,L}\left(t\right)+F_{Z,R}\left(t\right)}\;$$

where $\mathrm{Co}P_{L,y}\left(t\right)$ and $\mathrm{Co}P_{R,y}\left(t\right)$ are the AP positions from the left and right force plates, and $\mathrm{Co}P_{L,x}\left(t\right)$​ and $\mathrm{Co}P_{R,x}\left(t\right)$ are the corresponding ML positions. $F_{Z,L}\left(t\right)$ and $F_{Z,R}\left(t\right)$ are the vertical ground reaction forces from the left and right plates, respectively. The resulting CoP trajectory was then offset‐corrected by subtracting the mean position in each direction and segmented according to the previously identified reaching repetitions.

To quantify the displacement of the CoP, the path length ($PL_{\mathrm{CoP}}$) of each trajectory was computed in the horizontal plane. $PL_{\mathrm{CoP}}$ was used to quantify the total amount of displacement, reflecting the cumulative distance travelled by the CoP. This metric was computed for each repetition and normalized by the participant's height to allow for intersubject comparisons. $PL_{\mathrm{CoP}}$ was calculated as the cumulative Euclidean distance between consecutive sampling points in the AP and ML directions, based on the formulation originally proposed by Hufschmidt et al. (1980), and modified here to include height normalization:

(3) $$PL_{\mathrm{CoP}}=\frac{1}{h}\;\sum_{i=1}^{n-1}\sqrt{{\left(x_{i+1}-x_{i}\right)}^{2}+{\left(y_{i+1}-y_{i}\right)}^{2}}$$

where $\left(x_{i},y_{i}\right)$ represent the ML and AP coordinates, n is the number of samples, and h is the participant's height. For each condition, $PL_{\mathrm{CoP}}$ values across repetitions were then summarized by computing the median.

#### 2.5.3 EMG preprocessing

EMG data were high‐pass filtered using a fourth‐order, zero‐lag Butterworth filter with a cut‐off at 10 Hz to remove movement artifacts. After filtering, EMG data were full‐wave rectified, as indicated by previous studies (Boonstra & Breakspear, 2012; Halliday & Farmer, 2010; Myers et al., 2003; Ward et al., 2013), and concatenated across trials prior to the intermuscular coherence analysis. A sensitivity analysis using Hilbert‐derived envelopes and unrectified EMG signals was also conducted to exclude possible rectification effects (Supporting information, Figures S6–S7).

#### 2.5.4 Intermuscular coherence analysis

The magnitude‐squared coherence between EMG signals recorded from pairs of muscles was calculated to assess the frequency domain coupling between both bilateral and unilateral EMG signals for each reaching task. Bilateral EMG coherence was estimated between homologous muscles of the dominant and non‐dominant limbs, specifically the TA, GastM and Sol. Unilateral coherence was estimated between intra‐limb muscle pairs, TA–GastM, GastM–Sol, ES–GastM, ES–Sol and ES–TA, on both the dominant and non‐dominant sides. In this context, the term ‘dominant’ refers to the body side corresponding to the participant's dominant upper limb, which was used to perform the unilateral and lateral reaching tasks. This distinction was included to assess potential side‐specific modulation related to the functional involvement of the dominant limb during task execution.

A surrogate data analysis, involving the cross‐subject combination of EMG signals, was also employed to confirm that observed coherence between ES and ankle postural muscles (TA, GastM and Sol) reflected correlated neural inputs (Supporting information, Figure S4). Coherence was estimated using Welch's method with a Hanning window of 2048 samples and 75% overlap, yielding a frequency resolution of 0.98 Hz (Kattla & Lowery, 2010). For each EMG signal pair, *x*(*t*) and *y*(*t*), the magnitude squared coherence at frequency *f* was estimated as

(4) $$C_{xy}\;\left(f\right)=\frac{{\left|S_{xy}\left(f\right)\right|}^{2}}{S_{xx}\left(f\right)S_{yy}\left(f\right)}\;$$

where *S_xy_* (*f*) is the cross spectrum and *S_xx_* (*f*) and *S_yy_* (*f*) are the auto spectra of *x*(*t*) and *y*(*t*). The value of *C_xy_* (*f*) ranges from 0 to 1, where a value of 1 indicates a perfect linear relationship between *x*(*t*) and *y*(*t*), while a value of 0 indicates no linear relationship between the two signals at that frequency. For non‐overlapping segments with taper, a coherence estimate is typically deemed significant at P<α if the magnitude squared coherence exceeds $Z=1-\alpha^{1/(L-1)},$ where *L* represents the total number of disjoint segments utilized for calculating the coherence (Rosenberg et al., 1989) and *a* = 0.05, which corresponds to a confidence interval above 95%. To account for overlapping segments with taper, the significance level was modified according to the method described by Welch (1967) and adapted by Terry & Griffin (2008):

(5) $$Z_{\mathrm{ovlp}}=1-\alpha^{1/\left(wL^{*}-1\right)}\;$$

(6) $$L^{*}=\mathrm{floor}\left[\frac{\left(L-1\right)}{\left(1-\mathrm{ovlp}\right)}\right]+1\;$$

where the variable ovlp is the percentage of overlap between segments, while L^*^ denotes the count of overlapping segments. The weighting factor, *w*, depends on the degree of overlap and taper type. For tapered segments, the calculation of *w* follows the method outlined by Kattla & Lowery (2010). To reduce the probability of detection of spurious or ‘false’ coherence, the probability of detection $P_{D}$ was also determined using the approach described by Carter (1977). This quantity represents the probability that the estimated coherence $\hat{C}$ exceeds the threshold $Z_{\mathrm{ovlp}}$, given a true underlying coherence value *C*:

(7) $$P_{D}=\int_{Z_{\mathrm{ovlp}}}^{1}p\left(\hat{C}|L^{*},C\right)d\hat{C}=1-P\left(\hat{C}\leq Z_{\mathrm{ovlp}}|L^{*},C\right)\;$$

where $p\left(\hat{C}|L^{*},C\right)d\hat{C}$ is the conditional probability density function of the estimated coherence. The cumulative distribution function $P(\hat{C}\leq Z_{\mathrm{ovlp}}|L^{*},C)$ expresses the probability that the estimated coherence does not exceed the threshold, for a given true coherence *C* and number of overlapping segments $L^{*}$. Accordingly, coherence at a given frequency was considered statistically significant only if two conditions were met: (1) $\hat{C}>Z_{\mathrm{ovlp}}$, and (2) the probability of detecting such a coherence, $P_{D}$, exceeded 0.95 (Lowery et al., 2007). This dual criterion ensured both statistical significance and adequate detection power. The EMG–EMG coherence values that met these two criteria were then integrated within each of the delta (0–5 Hz), alpha (8–12 Hz), beta (13–35 Hz) and low gamma (35–60 Hz) frequency bands.

#### 2.5.5 Co‐contraction index

Co‐contraction was estimated using a modified version of the co‐contraction index (CCI) originally proposed by Rudolph et al. (2000), introducing temporal normalization to allow direct comparison across subjects. The EMG signals were first band‐pass filtered from 30–450 Hz using a 100th‐order finite impulse response (FIR) filter applied with zero‐phase distortion, to remove movement artifacts and high‐frequency noise. The signals were then full‐wave rectified, and the linear envelope extracted using a fourth‐order low‐pass Butterworth filter with a cutoff frequency of 10 Hz, applied with zero‐phase distortion. To ensure consistency with the coherence analysis, which was computed over concatenated repetitions, EMG envelopes were amplitude‐normalized using the median of the maximum values observed for each repetition and each muscle. The CCI was then computed on these globally normalized signals, allowing for direct comparison with coherence values derived from the same time window.

The CCI was computed using the following discrete‐time, duration‐normalized equation:

(8) $$\mathrm{CC}I_{\mathrm{norm}}=\frac{1}{T}\;\sum_{i=1}^{N}\left[\frac{\mathrm{EMG}S_{i}}{\mathrm{EMG}L_{i}}\;\cdot \;\left(\mathrm{EMG}S_{i}+\mathrm{EMG}L_{i}\right)\right]\;\cdot \;\Delta t$$

where EMGS is the activity of the less‐active muscle at each time point, EMGL is the activity of the more active muscle, Δt is the time interval between samples, and T is the total duration of the FR repetition. This sample‐by‐sample formulation yields a time‐averaged estimate of co‐contraction intensity. $\mathrm{CC}I_{\mathrm{norm}}$ was assessed between the TA and the GastM antagonist muscles and calculated separately for the dominant and non‐dominant sides.

### 2.6 Statistical analysis

Linear mixed‐effects models were applied to evaluate the influence of frequency band, task, dominance and muscle pair on coherence values. These factors and their interactions were incorporated as fixed effects, while participant was considered as a random factor to account for variability among subjects. Distinct models were developed for unilateral and bilateral conditions. *Post hoc* pairwise comparisons were carried out on the estimated marginal means, applying Bonferroni correction to adjust for multiple comparisons. Comparisons were performed within combinations of the relevant fixed factors, and the resulting *P*‐values were utilized to determine statistical significance. *P*‐values were presented using heatmaps organized by the relevant combinations of task, muscle pair, dominance and frequency band. *P*‐values below 0.05 were deemed statistically significant.

Correlation analyses were performed to investigate the relationships between intermuscular coherence and two outcome measures: the $\mathrm{CC}I_{\mathrm{norm}},$ which reflects neuromuscular coordination, and the $PL_{\mathrm{CoP}},$ which serves as an indicator of postural stability . The analyses were carried out independently for each frequency band (delta, alpha, beta and low gamma), task condition (biFR, uniFR, LR), and configuration (unilateral or bilateral). The Shapiro–Wilk test was used to evaluate the normality of coherence and each performance measure; based on those results, either Pearson's or Spearman's correlation coefficient was calculated. All obtained *P*‐values were corrected for multiple comparisons using the Bonferroni method and reported separately according to dominance (for $\mathrm{CC}I_{\mathrm{norm}}$) or configuration (for CoP).

The effect size of statistically significant differences was determined using Hedges's *g* statistic (Hedges, 1981). According to this measure, *g* values below 0.2 are considered small, those around 0.5 indicate medium effects and values above 0.8 represent large effects.

Statistical analyses were performed using MATLAB's Statistical and Machine Learning Toolbox (MATLAB version R2022b, The MathWorks Inc., Natick, MA, USA) and RStudio (version 2025.05.0+496, Posit Software, PBC, Boston, MA, USA).

## 3 RESULTS

### 3.1 Task‐related differences in intermuscular coherence

Overall, task‐dependent modulation of intermuscular coherence was observed between bilateral lower‐limb, unilateral lower‐limb, and trunk–limb muscle pairs in the delta, beta and low gamma bands.

### 3.2 Bilateral muscle pairs

**FIGURE 2:**
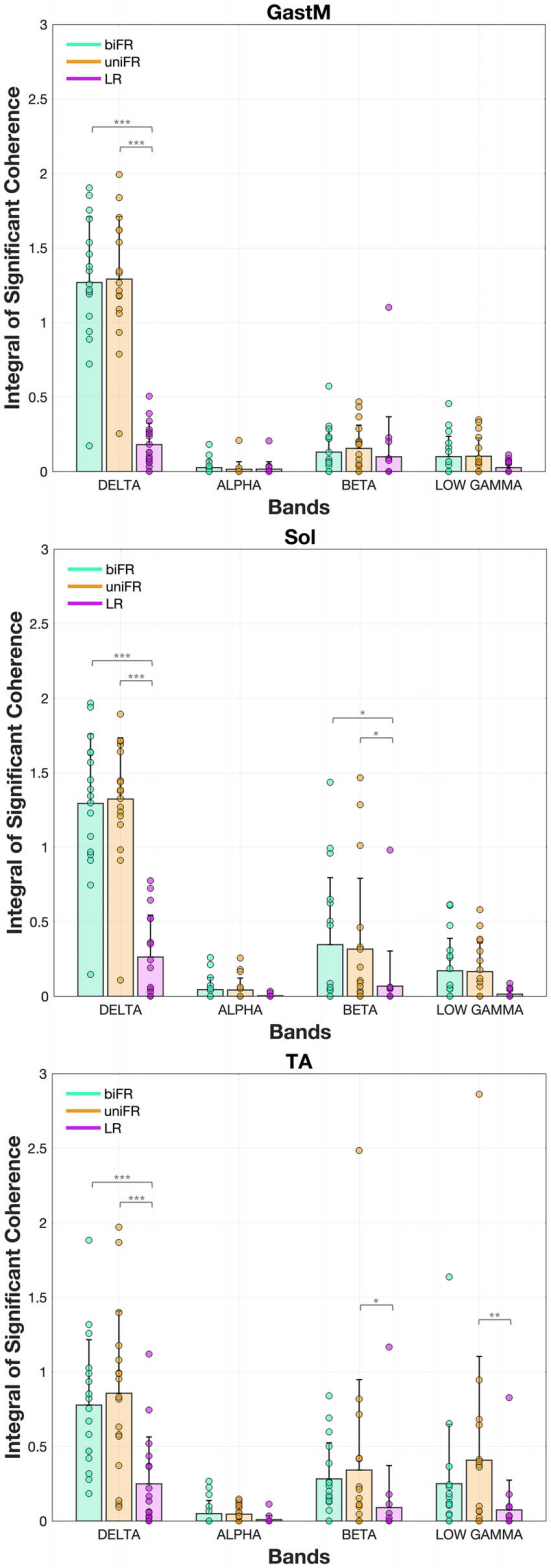
Mean values ± standard deviation (with individual data points overlaid) for bilateral muscle pairs of the integral of the coherence lying above the 5% significance threshold and satisfying the detection probability criterion, across the delta, alpha, beta and low gamma frequency bands, averaged over all subjects. Individual points are depicted for each participant (*n* = 17). Asterisks indicate significant differences across tasks (*P < 0.05; **P < 0.01; ***P < 0.001). Exact *P*‐values are reported in Supporting information, Figure S1.

Figure 2 shows the integral of the significant coherence in each frequency band, capturing the extent of task‐dependent modulation for each bilateral muscle pair. In the delta band, coherence was significantly higher during both biFR and uniFR compared to LR across all bilateral muscle pairs (GastM, Sol, TA), with all comparisons yielding *P* < 0.0001 (*g* = 1.765–3.712). In the beta band, coherence was greater in uniFR compared to LR for Sol (*P* = 0.0462, *g* = 0.834) and TA (*P* = 0.0433, *g* = 0.842), and in biFR compared to LR for Sol (*P* = 0.0208, *g* = 0.929). In the low gamma band, a task‐related difference emerged only for the TA, showing higher coherence in uniFR than LR (*P* = 0.00360, *g* = 1.115), with no significant differences observed for the GastM (*P* = 1.00) or Sol (*P* = 0.420). Alpha‐band coherence remained low across all muscle pairs and was unaffected by task.

**FIGURE 3:**
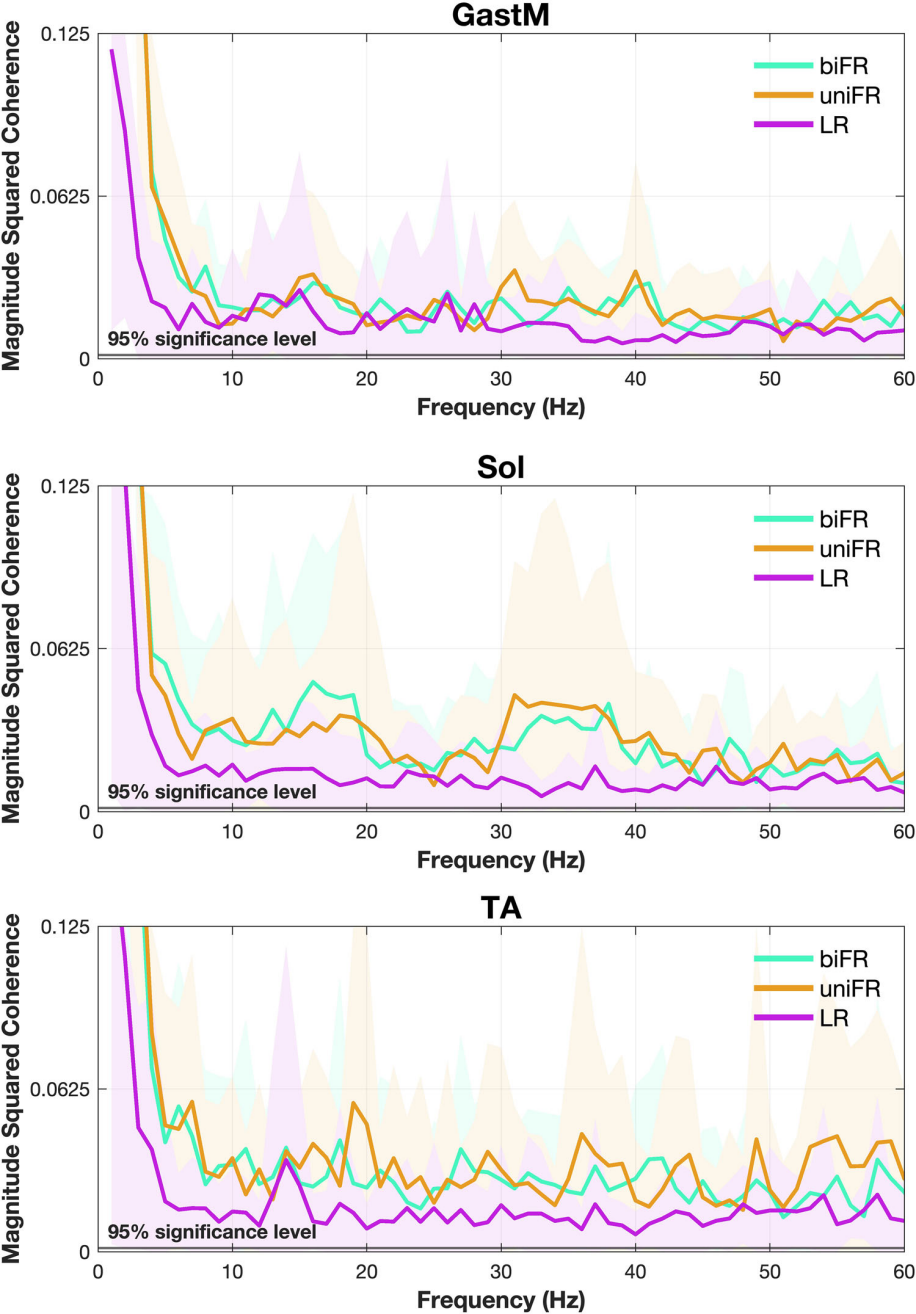
Average bilateral magnitude squared coherence across participants. The *y*‐axis was limited to 0.125 to enhance the visibility of condition‐related differences in coherence. The shaded area represents the standard deviation.

The results of the statistical analysis of the effect of task on coherence within each frequency band for the bilateral muscle pairs are presented in Supporting information, Figure S1. The bilateral intermuscular coherence spectra averaged across all subjects are presented in Figure 3.

### 3.3 Unilateral limb–limb muscle pairs

**FIGURE 4:**
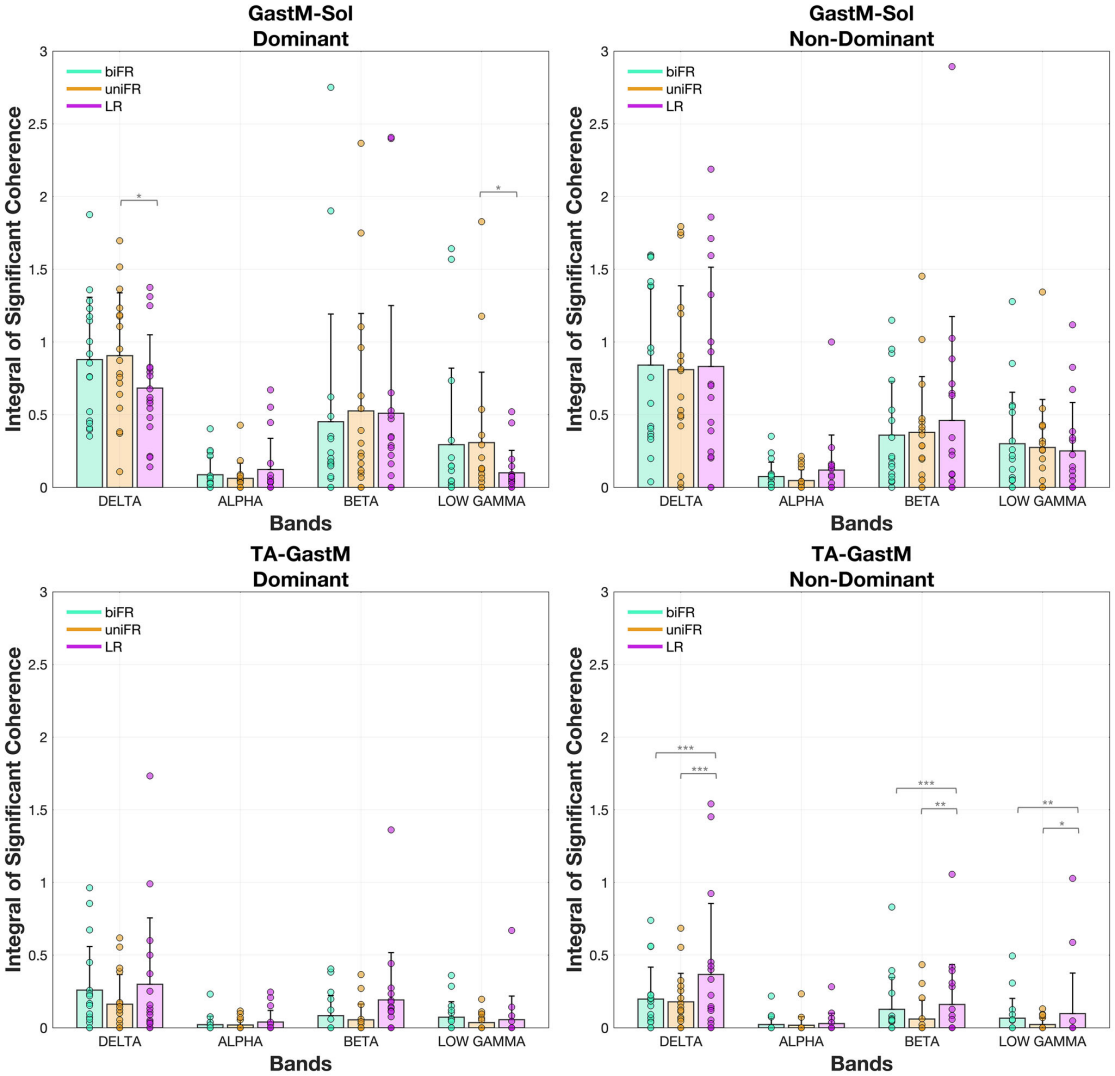
Mean values ± standard deviation (with individual data points overlaid) for unilateral muscle pairs of the integral of the coherence lying above the 5% significance threshold and satisfying the detection probability criterion, across the delta, alpha, beta and low gamma frequency bands, averaged over all subjects. Individual points are depicted for each participant (*n* = 17). Asterisks indicate significant differences across tasks (*P < 0.05; **P < 0.01; ***P < 0.001). Exact *P*‐values are reported in Supporting information, Figure S1.

The integral of the significant coherence between unilateral muscle pairs within each frequency band is presented in Figure 4. In the delta band, coherence showed distinct task‐related modulations for specific muscle pairs. For the GastM–Sol pair on the dominant side, coherence was significantly higher in uniFR compared to LR (*P* = 0.0226, *g* = 0.918). In contrast, the TA–GastM pair on the non‐dominant side consistently showed greater coherence during LR compared to both uniFR and biFR, with significant differences observed across the delta (*P* < 0.001, *g* = 2.601 and *g* = 2.521, respectively), beta (*P* < 0.001, *g* = 1.308, and *P* = 0.00770, *g* = 1.036, respectively) and low gamma bands (*P* = 0.00740, *g* = 1.040, and *P* = 0.0368, *g* = 0.860, respectively). No significant task‐related modulation was observed in the alpha band for any unilateral muscle pair.

**FIGURE 5:**
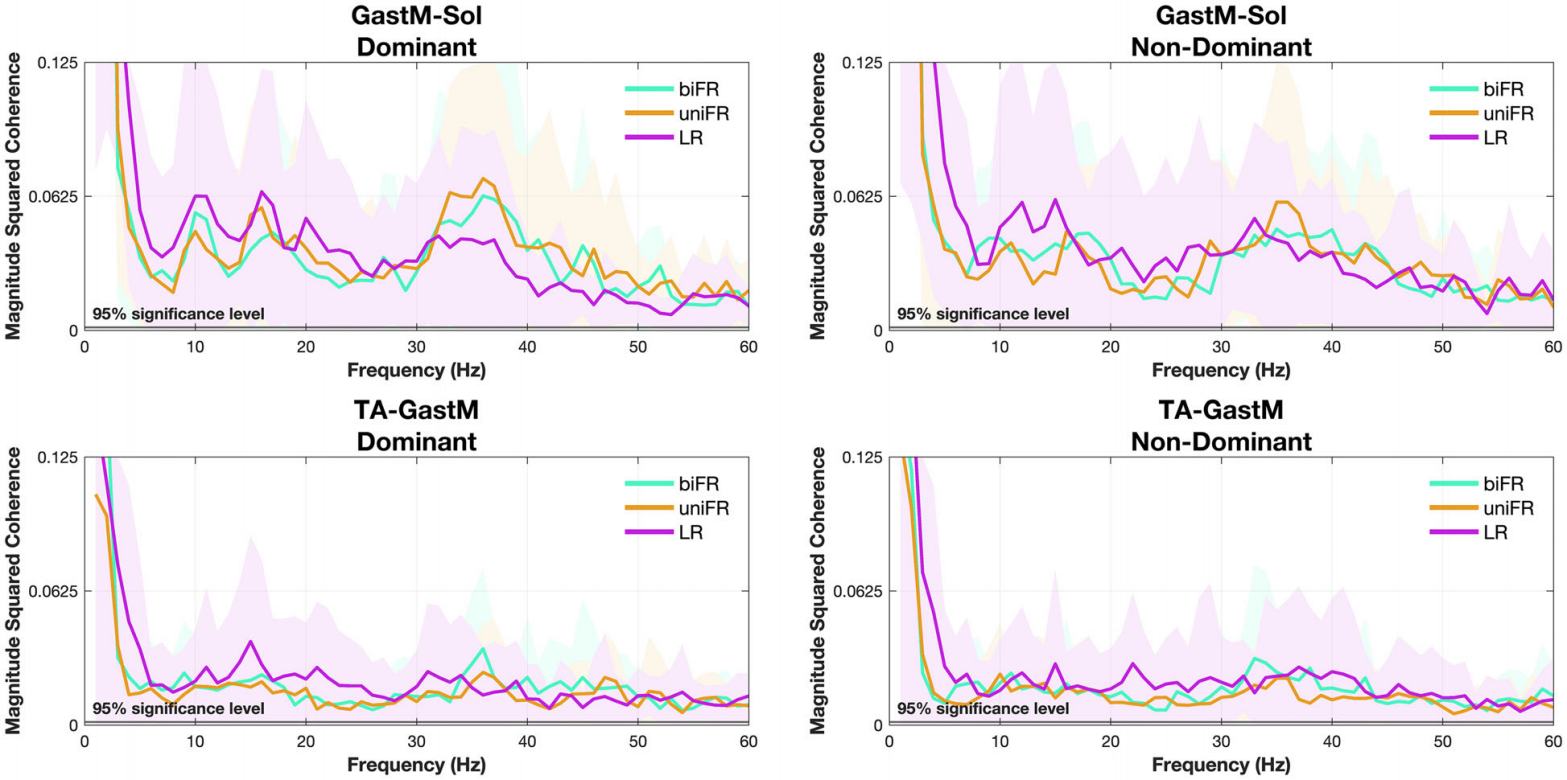
Average unilateral magnitude squared coherence across participants. Blue, red, and yellow represent the biFR, uniFR and LR tasks, respectively. The *y*‐axis was limited to 0.125 to enhance the visibility of condition‐related differences in coherence. The shaded area represents the standard deviation.

The results of the statistical analysis of the effect of task on coherence within each frequency band for the unilateral limb–limb muscle pairs are presented in Supporting information, Figure S1. The bilateral intermuscular coherence spectra averaged across all participants are presented in Figure 5.

### 3.4 Unilateral trunk–limb muscle pairs

**FIGURE 6:**
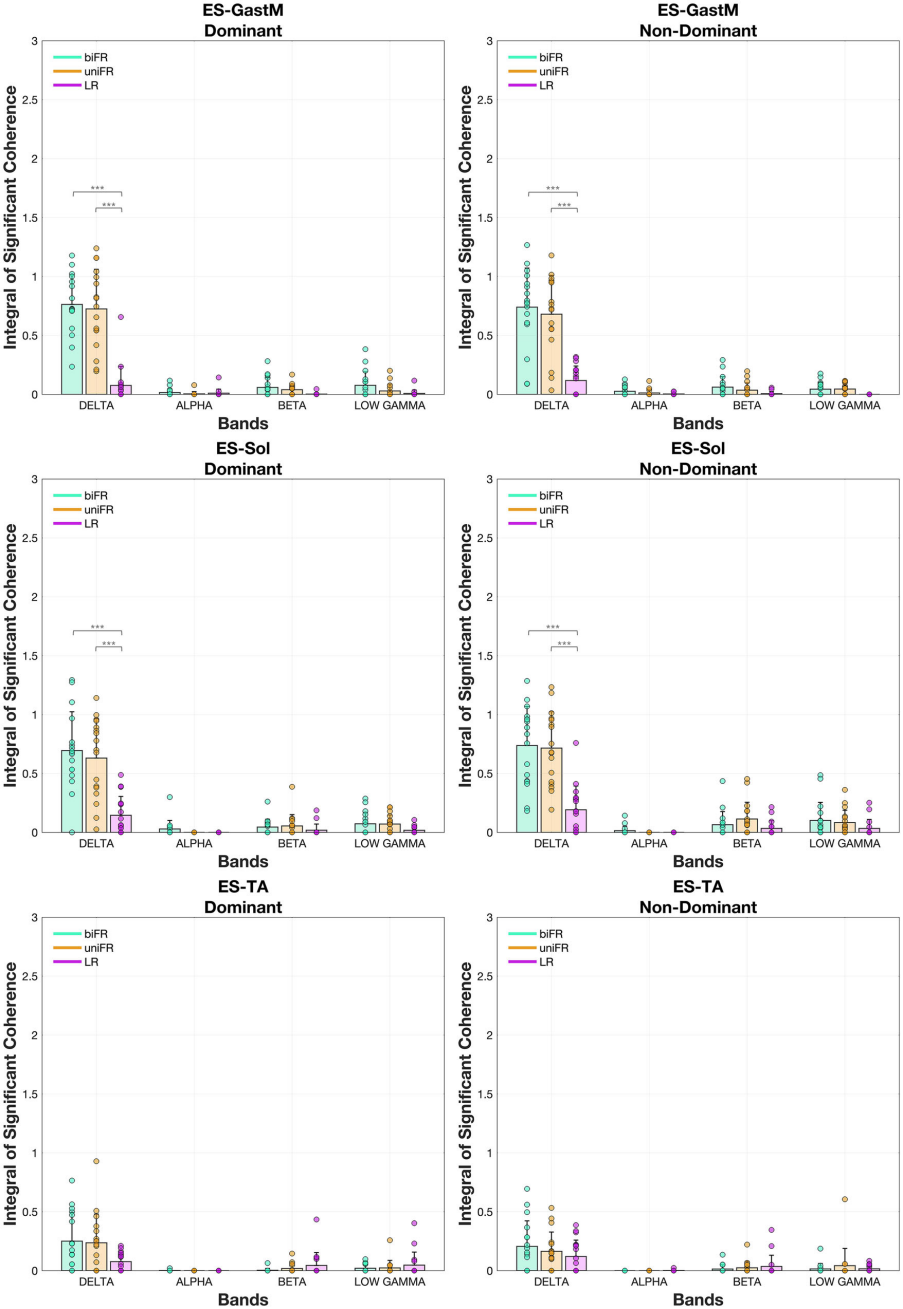
Mean values ± standard deviation (with individual data points overlaid) for unilateral ES‐ankle muscle pairs of the integral of the coherence lying above the 5% significance threshold and satisfying the detection probability criterion, across the delta, alpha, beta and low gamma frequency bands, averaged over all subjects. Individual points are depicted for each participant (*n* = 17). Asterisks indicate significant differences across tasks (*P < 0.05; **P < 0.01; ***P < 0.001). Exact *P*‐values are reported in Supporting information, Figure S1.

The integral of the significant coherence between the unilateral trunk and limb muscle pairs involving the ES muscle examined is presented in Figure 6 for each task and frequency band. In the delta band, task‐related modulation was evident: coherence for ES–GastM and ES–Sol was significantly higher during biFR and uniFR compared to LR (*P* < 0.001, *g* = 2.001–2.833, both sides), while no differences were observed across tasks for ES–TA (*P* = 1.00). No task‐related effects emerged in the beta, low gamma or alpha bands for any ES‐involved pairing.

**FIGURE 7:**
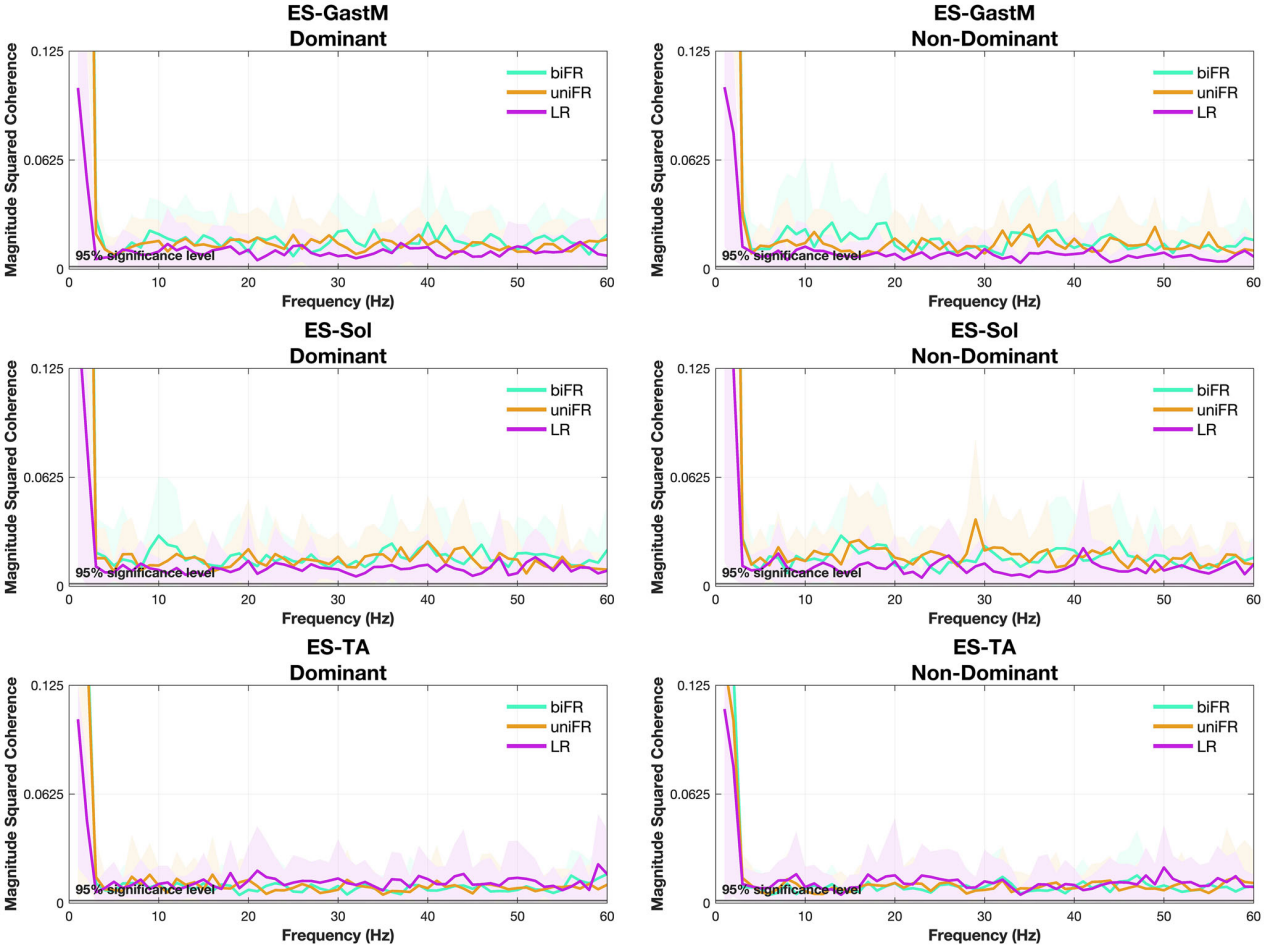
Average unilateral ES–ankle muscle magnitude squared coherence across participants. Blue, red and yellow represent the biFR, uniFR and LR tasks, respectively. The *y*‐axis was limited to 0.125 to enhance the visibility of condition‐related differences in coherence. The shaded area represents the standard deviation.

The results of the statistical analysis of the effect of task on coherence within each frequency band for the unilateral trunk–limb muscle pairs are presented in Supporting information, Figure S1. Intermuscular coherence spectra between the trunk and lower limb muscles on both the dominant and non‐dominant sides, averaged across all subjects, are presented in Figure 7.

### 3.5 Muscle pair‐related differences in intermuscular coherence

In addition to the task‐dependent differences in intermuscular coherence, significant differences were observed between muscle groups in the delta, beta and low gamma bands. During forward reaching (biFR and uniFR), delta‐band coherence was significantly higher for the bilateral GastM and Sol muscle pairs compared to the TA (*P* < 0.001, *g* = 1.450–1.724). No significant differences between bilateral muscle pairs were observed in the alpha or beta bands. In the low gamma band, the TA pair exhibited higher coherence than the GastM during uniFR (*P* = 0.00910, *g* = 1.021).

In the delta band, the agonist GastM–Sol pair exhibited significantly higher coherence than the antagonist TA–GastM pair across all tasks and on both limb sides (*P* < 0.001, *g* = 1.583–3.064), with the effect being especially pronounced during biFR and uniFR (Figure 4). When comparing trunk–limb combinations, both ES–GastM and ES–Sol exhibited higher coherence than ES–TA during biFR and uniFR (*P* < 0.001, *g* = 1.627–2.273) (Figure 6). There were no significant differences between ES–GastM and ES–Sol in any task or side (*P* = 1.00). When comparing limb–limb to trunk–limb pairings, GastM–Sol showed higher coherence than both ES–GastM and ES–Sol during LR (*P* < 0.001, *g* = 2.217–2.941), while the latter two had higher coherence than TA–GastM in uniFR and biFR (*P* < 0.001, *g* = 1.794–2.315), but lower coherence in LR on the non‐dominant side (*P* < 0.001, *g* = −2.852 and *g* = −2.545, respectively). Coherence for ES–TA was consistently lower than that of GastM–Sol across all tasks and sides (*P* < 0.001, *g* = −2.928 to −2.498).

In the beta band, GastM–Sol coherence on the dominant side was significantly higher than that of all trunk–limb muscle pairs examined (*P* < 0.001, *g* = 1.620–2.090). This difference was also present on the non‐dominant side across tasks: in LR (*P* < 0.001, *g* = 1.751–1.873), in biFR (e.g., vs. ES–GastM *P* = 0.00360, *g* = 1.226; ES–Sol *P* = 0.00420, *g* = 1.212; ES–TA *P* < 0.001, *g* = 1.421) and in uniFR (e.g., vs. ES–GastM *P* < 0.001, *g* = 1.410; ES–Sol *P* = 0.0154, *g* = 1.088; ES–TA *P* < 0.001, *g* = 1.457). GastM–Sol also showed higher coherence than the antagonist TA–GastM pair in biFR (*P* < 0.001, *g* = 1.517, dominant side), uniFR (*P* < 0.001, *g* = 1.943, dominant side; *P* = 0.00140, *g* = 1.308, non‐dominant side) and LR (*P* = 0.00140, *g* = 1.311, dominant side). No significant differences were observed between the ES–involved pairs themselves (all *P* = 1.00). During LR on the non‐dominant side, TA–GastM coherence was significantly higher than ES–GastM (*P* < 0.001, *g* = 1.533), ES–Sol (*P* < 0.001, *g* = 1.418) and ES–TA (*P* < 0.001, *g* = 1.411).

In the low gamma band, GastM–Sol coherence was higher than that of TA–GastM in uniFR on both the dominant (*P* = 0.0112, *g* = 1.120) and non‐dominant (*P* = 0.0246, *g* = 1.040) sides. In line with beta band findings, TA–GastM also showed greater coherence than ES–GastM (*P* = 0.00980, *g* = 1.132), ES–Sol (*P* = 0.0388, *g* = 0.992) and ES–TA (*P* = 0.0191, *g* = 1.066) during LR on the non‐dominant side. GastM–Sol was higher than ES–TA in biFR (*P* = 0.0106, *g* = 1.124, dominant; *P* = 0.00630, *g* = 1.175, non‐dominant), in uniFR (*P* = 0.00650, *g* = 1.172, dominant) and in LR (*P* = 0.0480, *g* = 0.969, non‐dominant). GastM–Sol coherence was also greater than ES–GastM in uniFR (*P* = 0.00820, *g* = 1.150, dominant), and in biFR (*P* = 0.0216, *g* = 1.054) and LR (*P* = 0.0259, *g* = 1.035) on the non‐dominant side.

No significant differences were observed in the alpha band between any limb–limb or trunk–limb pairings.

Statistical results are reported in the supplementary information in Supporting information, Figure S2.

### 3.6 Limb dominance‐related differences in intermuscular coherence

TA–GastM coherence was statistically higher during LR on the non‐dominant side than the dominant side for the delta (*P* < 0.001, *g* = 2.103), beta (*P* = 0.0252, *g* = 0.768) and low gamma (*P* = 0.0840, *g* = 0.905) bands. No other significant differences were detected between sides for any other muscle pair or task examined.

Details of the statistical results are presented in the supplementary information in Supporting information, Figure S3.

### 3.7 Correlation of coherence with postural stability and co‐contraction index

**FIGURE 8:**
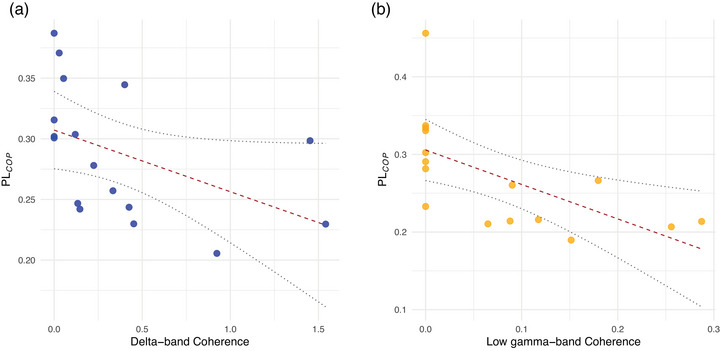
Scatter plots showing the associations between intermuscular coherence and the path length of the centre of pressure ($PL_{\mathrm{CoP}}$). (a) Delta‐band coherence of the TA–GastM pair on the non‐dominant side during LR. (b) Low gamma‐band coherence of the ES–Sol pair on the dominant side during biFR. Dotted lines indicate the 95% confidence intervals of the regression.

On the non‐dominant side, a significant negative correlation was observed between delta‐band coherence of the TA–GastM pair and $PL_{\mathrm{CoP}}$ during LR (*P* = 0.0456, *r* = −0.707, *g* = −1.895), as illustrated in Figure 8a. Additionally, on the dominant side, low gamma‐band coherence between the ES–Sol pair showed a significant negative correlation with $PL_{\mathrm{CoP}}\;$ during biFR (*P* = 0.0147, *r* = −0.770, *g* = −2.289) (see Figure 8b). No other significant correlations were observed across frequency bands, tasks, and muscle pairings on either the dominant or non‐dominant side (see Supporting information, Figure S5).

**FIGURE 9:**
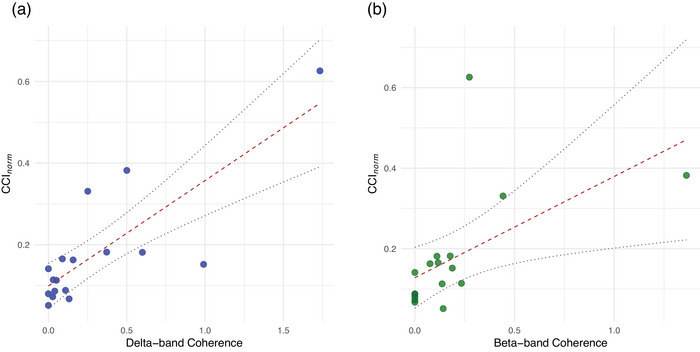
Scatter plots showing the associations between intermuscular coherence and the normalized co‐contraction index ($\mathrm{CC}I_{\mathrm{norm}}$). (a) Delta‐band coherence on the dominant side during LR. (b) Beta‐band coherence on the dominant side during LR. Dotted lines indicate the 95% confidence intervals of the regression.

On the dominant side, a significant positive correlation was found between delta‐band coherence and $\mathrm{CC}I_{\mathrm{norm}}$ during LR (*P* = 0.00730, *r* = 0.745, *g* = 2.117) (Figure 9a), as well as between beta‐band coherence and $\mathrm{CC}I_{\mathrm{norm}}$ in the same condition (*P* = 0.0319, *r* = 0.680, *g* = 1.762) (Figure 9b).

## 4 DISCUSSION

Consistent with our hypotheses, intermuscular coherence varied according to both the direction of reaching and the muscle combinations involved, demonstrating that neural coupling is flexibly tuned to the biomechanical requirements of each task. As participants transitioned from quiet standing to destabilized postures in the sagittal and frontal planes, the CNS adjusted the distribution and synchronization of shared neural inputs to coordinate trunk and lower‐limb muscles in a directionally appropriate way. In parallel, functionally synergistic muscles showed synchronized activity, confirming the presence of a common neural drive that contributes to postural stability.

During forward reaching tasks (biFR and uniFR), coherence was significantly higher in the delta band, both among bilateral homologous muscles (TA, GastM, Sol) and between trunk–limb pairs of the posterior chain (ES–GastM, ES–Sol). Additionally, the synergistic ankle plantar‐flexors (GastM–Sol) exhibited greater delta‐band coherence compared to the antagonistic muscles (TA–GastM). The delta‐band component is generally considered an index of the common neural drive to muscles, particularly during tasks involving low‐force, static contractions and slow, steady force production such as postural maintenance (Borzuola et al., 2025; Farina & Negro, 2015). These low‐frequency oscillations are thought to originate primarily from subcortical structures rather than the corticospinal system (Boonstra et al., 2009; De Luca & Erim, 1994; Kamen & & De Luca, 1992) and can also be modulated by shared afferent feedback and other subcortical mechanisms (Boonstra & Breakspear, 2012; Yamanaka et al., 2023). Our findings suggest a predominance of subcortical common drive, which promotes coordinated low‐frequency activation of muscles that contribute to sagittal stability. Such organization is consistent with prior studies showing robust delta band among bilateral homologous muscle pairs and unilateral plantar flexors during static standing tasks (Boonstra et al., 2009; Mochizuki et al., 2006; Obata et al., 2014; Watanabe et al., 2018a, 2018b). The elevated coherence in trunk–limb extensors pairs indicates the integration of trunk and lower‐limb extensors into a posterior‐chain synergy, primarily driven by subcortical networks that stabilize the body in the sagittal plane. This finding supports the conclusions of Noé et al. (2017), suggesting that postural instability promotes functional integration between trunk and lower‐limb extensors, supporting the idea that the CNS dynamically integrates proximal and distal muscles into unified coordination patterns when postural constraints increase. Furthermore, consistent with reports by Danna‐Dos‐Santos et al. (2014), the significant coherence between synergistic muscles in the posterior chain (e.g., GastM–Sol, ES–GastM) reflects a simplification of motor control by the CNS, which uses common neural inputs to stabilize posture through multi‐muscle synergies. Notably, surrogate analysis (Supporting information, Figure S4) confirmed that the observed coherence between ES and ankle muscles was not attributable to spurious coupling, but rather reflects true functional synchronization, underscoring the neural origin of these synergies.

Additionally, in all tasks, the synergistic plantar‐flexor GastM–Sol pair consistently showed higher coherence than the antagonist TA–GastM pair. The limited modulation of beta‐ and low gamma‐band coherence across tasks suggests that the GastM–Sol coupling is largely stable and reflects the automatic, subcortically mediated nature of the plantar‐flexor synergy. However, coherence within the GastM–Sol pair tended to be greater in the dominant limb during lateral reaching (LR), where the mechanical demands for fine balance adjustments increase. This enhancement likely reflects a cortical contribution to synergy control, consistent with evidence that beta‐band coherence between synergistic muscles increases during complex standing tasks (Nandi et al., 2019; Nojima et al., 2020; Watanabe et al., 2018a, 2018b). Beta‐band coherence has been associated with corticospinal common drive, particularly in dynamic or voluntary tasks such as gait (Jensen et al., 2018), bipedal squatting (Kenville et al., 2020) and forward leaning (Watanabe et al., 2018a). Fisher et al. (2012) further emphasized that intermuscular coherence in the beta range depends on the functional integrity of the corticospinal tract, which plays a central role in voluntary motor control (Welniarz et al., 2017). Furthermore, recent evidence has shown that beta‐band coherence reflects not only corticospinal drive but also cortical‐level coordination of functionally synergistic muscles (Laine & Valero‐Cuevas, 2017; Reyes et al., 2017), supporting the idea that higher coherence in these frequencies may reflect active cortical control over multi‐muscle synergies. In addition to the beta band, coherence in the low gamma band also showed the same pattern. Indeed, not only the beta band but also the low gamma band has been associated with transcortical pathways (Conway et al., 1995; Fisher et al., 2012), and their joint presence suggests cortical involvement in the coordination of postural muscles during upright reaching. In this context, the elevated GastM–Sol coherence on the dominant side may represent a voluntary, transcortically mediated modulation that fine‐tunes synergistic activation to maintain stability during asymmetric postural demands. Thus, while the plantar‐flexor synergy operates primarily under subcortical common drive, it remains adaptively upregulated by cortical mechanisms when task complexity or directional instability increases.

Moreover, during lateral reaching, an asymmetric reorganization of coherence between limbs indicates a redistribution of neural control across subcortical and cortical pathways. Enhanced intra‐limb coherence between plantar‐flexor synergists, as well as between agonist–antagonist muscle pair, suggests that the CNS reinforces local neuromuscular coupling to stabilize load‐bearing joints under asymmetric conditions. This is consistent with findings from Obata et al. (2014), who reported that both intra‐ and inter‐limb coherence are modulated based on the distribution of effort and task‐specific postural constraints. In the non‐dominant side, the antagonist TA–GastM displayed higher coherence across delta, beta and low gamma bands compared to both forward reaching conditions, as well as the TA–GastM pair on the dominant side. Higher delta‐band coherence in this limb was negatively correlated with $PL_{\mathrm{CoP}},$ suggesting that enhanced antagonist coupling improved postural stability, likely through increased ankle joint stiffness (Baratto et al., 2002). Such predominance of low‐frequency synchronization under medial–lateral instability supports previous evidence that antagonist coupling increases with postural challenge (Nandi et al., 2019; Nojima et al., 2020). In contrast, in the dominant side, coherence in TA–GastM did not show significant differences across tasks but was positively correlated with the $\mathrm{CC}I_{\mathrm{norm}}$ in both delta and beta bands. This suggests that joint stability was achieved via voluntary modulation of stiffness through shared neural input. Antagonist co‐contraction has been demonstrated to effectively enhance joint stiffness during motor tasks (Geertsen et al., 2013; Hansen et al., 2001), and in this scenario, it likely played a role in maintaining accuracy and control throughout the reaching movement without sacrificing postural stability. Together, these results confirm a clear asymmetry in neural control strategies between limbs. The non‐dominant limb, which was responsible for stabilizing the body under lateral load, relied on increased low‐frequency coherence, indicative of more automatic coordination. In contrast, the dominant limb employed a more refined and voluntary control through co‐contraction mechanisms, with neural coherence reflecting the modulation of joint stiffness rather than a generalized response to postural demand. Notably, TA–GastM coherence on the non‐dominant side exceeded that of all trunk–limb pairings across the same frequency bands during LR, reinforcing the importance of ankle‐level coordination in medial–lateral stability. Altogether, this side‐specific organization of neural control suggests the inter‐limb independence as a strategy to counter lateral instability, also supporting the dual role of coherence in both automatic and voluntary components of postural control. This interpretation is consistent with findings by Mochizuki et al. (2006), who demonstrated that standing postural control relies primarily on automatic, subcortical mechanisms rather than voluntary drive. Their results further suggest that low‐frequency modulations in muscle activity during quiet stance may be closely related to CoP dynamics, which is consistent with the negative correlation observed in the present study between delta‐band coherence and CoP path length in the non‐dominant limb. In contrast, the presence of beta‐band coherence in the dominant limb, especially in association with antagonist co‐contraction, may reflect a voluntary, cortically mediated strategy to modulate joint stiffness and enhance movement precision under postural constraint.

Finally, although alpha‐band coherence was consistently present across all conditions, it remained low and unaltered, in line with previous literature. Alpha‐band coherence is primarily associated with subcortical and spinal mechanisms (Baker & Baker, 2003; Conway et al., 1995; McAuley & Marsden, 2000), which may play a limited role in dynamic postural regulation in healthy young adults. Supporting this, Obata et al. (2014) reported alpha‐range coherence only in elderly individuals, while it was absent in younger participants, further suggesting that coherence in this frequency band may become more prominent under compensatory or age‐related neuromuscular strategies.

Several limitations should be acknowledged in the present study. First, our sample was restricted to healthy young adults, which limits the generalizability of the findings to older adults or clinical populations with known deficits in postural control. Previous studies have shown age‐related alterations in intermuscular coherence, particularly reductions in low‐frequency synchronization and increased reliance on alpha‐band activity (Mochizuki et al., 2006; Obata et al., 2014; Watanabe et al., 2018a), suggesting that ageing may reshape the neural strategies underlying multi‐muscle coordination. Future research should explore how age‐related changes in the nervous system affect frequency‐specific coherence patterns, especially in tasks that challenge balance in the frontal and sagittal planes. Moreover, the limited sample size should be acknowledged as a potential constraint, as it may have reduced the ability to detect smaller effects despite the consistent trends observed in the data. Second, the definition of frequency bands, particularly the beta range, was based on conventions adopted in recent literature, including the systematic scoping review by Yamanaka et al. (2023). However, discrepancies still exist across studies regarding the exact frequency boundaries, especially in the beta and low gamma ranges. These methodological differences could lead to variation in the detection and interpretation of coherence patterns and highlight the need for greater consensus on frequency band definitions in EMG–EMG coherence studies of motor control. Third, although we investigated multiple trunk–limb pairings, bilateral coherence between left and right ES was not analysed due to the potential risk of signal cross‐talk in surface EMG recordings of paraspinal muscles. This precludes us from making direct inferences about trunk‐level bilateral coordination, which may also contribute significantly to postural stability. Moreover, extending the analysis to a larger set of trunk and limb muscles could provide a more integrated view of bilateral and intersegmental coordination mechanisms underlying postural control. Finally, while intermuscular coherence provides valuable insight into the presence of shared neural inputs, it does not reveal their precise origin – whether cortical, subcortical or spinal. Future studies combining EMG coherence with neuroimaging or electrophysiological approaches, such as EEG or MEG, may help to disentangle the hierarchical contributions of central versus peripheral mechanisms to postural coordination. This would further clarify how different frequency bands relate to distinct levels of neural control and refine our understanding of the neurophysiological basis of upright motor behaviour.

In conclusion, our findings underscore the value of intermuscular coherence as a window into the CNS's organization of postural strategies and provide new insights into the role of neural synchronization in coordinating muscle activity during reaching tasks. Delta‐band coherence supports low‐frequency coupling for automatic stabilization, particularly under sagittal and lateral postural loads. Beta‐ and low gamma‐band coherence are commonly associated with corticospinal/transcortical contributions and thus are consistent with cortical involvement during unilateral or asymmetric conditions. However, it is important to acknowledge that intermuscular coherence alone cannot definitively identify the neural source, so any conclusions drawn should remain inferential. The CNS appears to flexibly organize intra‐ and inter‐limb strategies to meet task‐specific mechanical demands. Notably, the systematic modulation of coherence across tasks and muscle pairings suggests that muscle coordination is not merely a direct response to mechanical constraints but often reflects centrally guided strategies. In this sense, coherence analysis may offer a meaningful way to differentiate between functionally necessary muscle groupings and those shaped by shared neural control, providing insight into how the CNS builds and modulates synergies to meet postural demands.

## AUTHOR CONTRIBUTIONS

Imma Ceriello, Riccardo Borzuola, Valentina Camomilla and Andrea Macaluso developed and planned the research; Imma Ceriello and Riccardo Borzuola conducted the experiments; Imma Ceriello analyzed the data; Imma Ceriello and Madeleine Lowery interpreted the experimental results; Imma Ceriello created the figures; Imma Ceriello and Riccardo Borzuola drafted manuscript; Imma Ceriello, Riccardo Borzuola, Valentina Camomilla, Andrea Macaluso and Madeleine Lowery reviewed and revised the manuscript. All authors have read and approved the final version of this manuscript and agree to be accountable for all aspects of the work in ensuring that questions related to the accuracy or integrity of any part of the work are appropriately investigated and resolved. All persons designated as authors qualify for authorship, and all those who qualify for authorship are listed.

## CONFLICT OF INTEREST

None declared.

## FUNDING INFORMATION

None.

## Supporting information



Figures S1–S7.

## Data Availability

The data that support these conclusions can be requested from the corresponding author upon a reasonable request.
