# Supplementary material for: Task‐dependent intermuscular coherence between postural muscles during voluntary upright reaching
Source: Exp Physiol. 2026 Mar 10;111(4):2106–22. doi: 10.1113/EP093222 (PMC13140409; doi:10.1113/EP093222)
Supplement: Supplementary file 1 — Figures S1–S7. [file EPH-111-2106-s001.docx]

# SUPPORTING INFORMATION

**Figure S1**. Heatmaps of p-values for pairwise task comparisons across frequency bands, muscle pairs, and dominance sides. The left panel (**A**) shows results for bilateral coherence, and the right panel (**B**) for unilateral coherence. Significant differences (p < 0.05, Bonferroni corrected) are highlighted.

**Figure S2**. Heatmaps of p-values for pairwise muscle pair comparisons across frequency bands, tasks, and dominance sides. The left panel (**A**) shows results for bilateral coherence, and the right panel (**B**) for unilateral coherence. Significant differences (p < 0.05, Bonferroni corrected) are highlighted.

**

***Figure S3.*** *Heatmap of p-values for pairwise comparisons between dominant and non-dominant sides (DOMINANT – NON-DOMINANT), across frequency bands and muscle pairs. Each cell displays the p-value of the contrast for a given task and muscle pair. Significant differences (p < 0.05, Bonferroni corrected) are highlighted in green.*

**Surrogate-based validation of trunk-limb muscle pairs coherence**

For every muscle pair that included the ES (ES–TA, ES–GastM, and ES–Sol, on both the dominant and non-dominant sides), a surrogate coherence spectrum was estimated between the ES EMG signal from one participant and a corresponding ankle EMG signal randomly selected from another participant, guaranteeing that there was no shared neural influence. This procedure was repeated 17 times for each muscle pair to create a surrogate dataset that corresponded to the number of subjects recorded, facilitating a direct comparison with the experimental coherence estimates. To enhance the robustness of the surrogate analysis, this procedure was further extended by repeating the random pairing process 100 times for each muscle pair. Surrogate coherence spectra were calculated using the same spectral parameters that were used for the experimental coherence analysis. Statistical significance benchmarks were applied to each of the 100 surrogate coherence spectra, and the results were subsequently averaged to obtain a stable reference distribution for each muscle pair. This approach enabled the evaluation of whether the observed ES–ankle muscle coherence reflected true functional coupling or could instead be attributed to spurious synchrony.

***Figure S4.*** *Mean ± standard deviation surrogate coherence values for the ES–ankle muscle pairs with individual data points overlaid.*

**

***Figure S5.*** *Heatmap of the correlation coefficients (Spearman’s r) between intermuscular coherence and the path length of the center of pressure (PL_CoP_) are shown for each frequency band, task, and muscle pair. Panel* ***(A)*** *illustrates the correlations for the combined analysis for bilateral coherence, while panel* ***(B)*** *reports results for unilateral coherence, separately for the dominant and non-dominant sides. Each circle represents the correlation coefficient for a given task (biFR, uniFR, LR) and frequency band (delta, alpha, beta, low gamma), with color indicating the direction and strength of the association (green: positive; red: negative). Statistically significant correlations (p < 0.05) are highlighted with bold contours.*

**Assessment of Rectification Effects on Coherence Estimation**

To evaluate the influence of rectification on coherence estimation, we performed a sensitivity analysis based on two alternative preprocessing approaches: coherence computed from Hilbert-derived envelopes following the procedure of Myers et al. (2003), and coherence computed from unrectified EMG signals that were only high-pass filtered at 10 Hz prior to analysis. For each muscle pair and task, intermuscular coherence was estimated from either the analytic signal (Hilbert) or the unrectified high-pass filtered signal, thereby bypassing the rectification step. Representative results are presented in **Figures S6** and **S7**. Both approaches preserved the low-frequency features and task-dependent modulations observed in the rectified-signal analysis, with delta-band coherence remaining clearly expressed. This persistence supports the interpretation that the reported low-frequency coherence reflects genuine shared neural input rather than preprocessing artefacts.

***Figure S6.*** *Comparison between coherence estimated from Hilbert-derived envelopes, unrectified EMG signals, and rectified EMG signals. Panel (****A****) shows intermuscular coherence computed from Hilbert-derived envelopes as part of the rectification sensitivity analysis. Panel (****B****) shows coherence estimated from unrectified EMG signals that were only high-pass filtered at 10 Hz prior to analysis. Panel (****C****) reports the corresponding coherence estimates obtained from rectified EMG signals in the main analysis. Representative results from selected muscle pairs are shown.*

***Figure S7.*** *Power spectra for GastM during LR using rectified, unrectified, and Hilbert-derived envelope EMG.*
